# Problematic reliance on generative AI among university students: an I-PACE-informed PLS-SEM and fsQCA study

**DOI:** 10.3389/fpsyg.2026.1946110

**Published:** 2026-10-05

**Authors:** Guoqing Liu, Caisang Hou

**Affiliations:** 1 School of Physical Education, Minnan Normal University, Zhangzhou, China; 2 Faculty of Education, The University of Hong Kong, Hong Kong, Hong Kong SAR, China

**Keywords:** academic stress, fsQCA, I-PACE model, perceived usefulness, PLS-SEM, problematic reliance on generative AI, university students

## Abstract

Generative AI has become increasingly embedded in university students’ academic learning, offering immediate feedback, content generation, and problem-solving support. Alongside these benefits, repeated reliance has raised concerns about problematic dependence on AI-mediated academic support. Drawing on an I-PACE-informed framework, this cross-sectional study examined psychological and academic factors associated with learning-related problematic reliance on generative AI, defined as a non-clinical, self-reported pattern rather than a clinical diagnosis. Data were obtained from a non-probability online sample of 242 university students who had used generative AI for learning-related purposes. Partial least squares structural equation modeling (PLS-SEM) was used to examine associations among positive affect, generative-AI use self-efficacy, need for cognition, perceived usefulness, cognitive absorption, AI-based mood regulation, academic stress, and problematic reliance on generative AI. Fuzzy-set qualitative comparative analysis (fsQCA) was further used to identify configurations associated with high problematic-reliance membership and its set-theoretic complement. The PLS-SEM results showed that perceived usefulness, cognitive absorption, and academic stress were positively associated with problematic reliance on generative AI, whereas positive affect and AI-based mood regulation did not show significant direct associations. The fsQCA results indicated that no single condition was necessary for high problematic-reliance membership; instead, both outcome sets were associated with multiple configurations. The findings support a cautious distinction between problematic reliance, ordinary generative AI use, and technology acceptance, and they suggest potential implications for AI literacy and academic support in higher education.

## 1 Introduction

Generative artificial intelligence has rapidly entered higher education and has become part of university students’ everyday learning practices. Unlike conventional digital tools that mainly provide access to existing information, generative AI systems can respond to natural language prompts, generate text, summarize materials, explain concepts, provide feedback, and support iterative problem solving. Student-focused studies have shown that university students are already engaging with generative AI tools, evaluating their perceived benefits and challenges, and forming expectations about their value, cost, and appropriate use in academic work (Baek et al., 2024; Chan and Hu, 2023; Chan and Zhou, 2023). Recent research on generative AI literacy in higher education further suggests that students’ adoption and interaction with generative AI are closely connected with their ability to evaluate outputs and address ethical issues (Chen et al., 2025).

The same characteristics that make generative AI useful also create a distinctive form of educational risk. Because generative AI can participate directly in content generation, reasoning support, and task completion, students may not only use it to support learning but also begin to delegate thinking, writing, organizing, and decision-making processes to AI systems. Prior work has emphasized that large language models can create opportunities for learning support and personalization, while also raising concerns about unreliable outputs, overreliance, weakened critical engagement, assessment validity, and academic integrity (Cotton et al., 2024; Farrokhnia et al., 2024; Kasneci et al., 2023; Lo, 2023; Rudolph et al., 2023). These concerns indicate that the key issue is no longer only whether university students use generative AI, but how AI-supported learning may gradually develop into excessive reliance on generative AI.

In this study, problematic reliance on generative AI refers to a non-clinical, self-reported pattern of excessive learning-related reliance on generative AI. The four indicators capture perceived overuse, difficulty limiting use, interference with completing learning tasks independently, and unease or frustration when generative AI is unavailable. The construct is not treated as a clinical diagnosis and does not classify appropriate, supportive, or efficient AI use as problematic. For consistency with the existing dataset and statistical outputs, GA is retained solely as the statistical code for this construct. This distinction is consistent with research describing AI dependency as excessive use accompanied by psychological reliance rather than ordinary technology acceptance or continuance use (Zhang et al., 2024; Zhong et al., 2024).

Existing studies on generative AI in education have largely focused on technology acceptance, usage intention, perceived educational value, learning support, and academic integrity risks (Baek et al., 2024; Chan and Hu, 2023; Chan and Zhou, 2023; Chen et al., 2025; Cotton et al., 2024; Farrokhnia et al., 2024; Kasneci et al., 2023; Strzelecki, 2024). These studies clarify why students may adopt or value generative AI tools, but they do not fully explain why some students move from ordinary academic use to excessive reliance. Recent studies have begun to examine AI dependency in higher education. Zhang et al. (2024) examined the roles of academic self-efficacy, academic stress, and performance expectations in problematic AI use among university students, while Zhong et al. (2024) explored how personal attributes and psychological mechanisms shape AI dependency in Chinese higher education. However, there remains a need to explain how affective experiences, AI-related ability beliefs, cognitive dispositions, perceived tool value, deep engagement, AI-based Mood Regulation, and academic pressure jointly shape problematic reliance on generative AI.

The I-PACE model provides a suitable theoretical basis for this purpose. The model explains problematic technology-related behavior as the outcome of interactions among person-related characteristics, affective responses, cognitive evaluations, and execution or use processes (Brand et al., 2016, 2019, 2025). In the present study, Self-Efficacy and Need for Cognition represent person-related characteristics; Positive Affect reflects affective reinforcement during AI-assisted learning; Perceived Usefulness captures students’ cognitive evaluation of AI’s learning value; Cognitive Absorption reflects deep engagement during repeated AI interaction; Mood Regulation reflects AI-based emotional and academic stress regulation; and Academic Stress represents the learning-contextual pressure that may push students toward AI-supported coping. In this way, problematic reliance on generative AI can be examined as a psychological and contextual pattern rather than a simple consequence of technology exposure.

Methodologically, this study combines PLS-SEM and fsQCA. PLS-SEM is appropriate for estimating theoretically specified associations among latent constructs and for examining the average associations of individual antecedents (Hair et al., 2019, 2022). However, problematic reliance on generative AI may not be associated with a single linear and symmetrical pattern. Students with different psychological profiles may show similarly high problematic-reliance membership through different combinations of conditions. fsQCA is therefore used to examine configurational patterns, equifinality, and set-theoretic asymmetry (Fiss, 2011; Pappas and Woodside, 2021; Ragin, 2008; Schneider and Wagemann, 2012). Combining these two methods allows the study to address both “which variables matter on average” and “which combinations of conditions are associated with high problematic-reliance membership.”

This study makes three bounded contributions. First, it conceptually replicates and extends the teacher-focused I-PACE study of Du et al. (2026) in a university-student learning context, while adding academic stress as a contextual condition. Second, it uses an I-PACE-informed framework to organize personal, affective, cognitive, regulatory, and academic-pressure factors associated with problematic reliance on generative AI without claiming to test the full developmental process specified by I-PACE. Third, by combining PLS-SEM and fsQCA, it examines both average associations and configurational patterns. The findings may inform future work on AI literacy, academic support, and responsible generative AI use, although the cross-sectional design does not establish the causal effectiveness of particular interventions (Chen et al., 2025; Long and Magerko, 2020; Ng et al., 2021).

## 2 Literature review and hypotheses

### 2.1 Problematic reliance on generative AI and the I-PACE model

Generative AI has become increasingly integrated into university learning. It can support text generation, knowledge explanation, feedback revision, problem solving, and task advancement through natural language interaction. Student-focused studies have documented university students’ perceptions, use patterns, perceived value, and concerns regarding generative AI in higher education (Baek et al., 2024; Chan and Hu, 2023; Chan and Zhou, 2023; Chen et al., 2025). These characteristics make generative AI valuable in learning, but they also create the possibility that students may gradually rely on AI outputs and AI-mediated academic support.

Building on this distinction, the present study treats problematic reliance on generative AI as a non-clinical, learning-related pattern indicated by perceived overuse, difficulty limiting use, interference with independent task completion, and negative reactions when generative AI is unavailable. This operationalization is narrower than a clinical behavioral-addiction diagnosis and should not be interpreted as a prevalence measure or diagnostic classification. GA is retained only as the statistical code in the original dataset, model outputs, path notation, and item labels.

The I-PACE model provides the main theoretical orientation for this study. It proposes that problematic technology-related behavior develops through interactions among person-related characteristics, affective responses, cognitive evaluations, and execution-related processes (Brand et al., 2016, 2019, 2025). In the present I-PACE-informed model, generative-AI use self-efficacy (SE) and need for cognition (NC) represent person-related characteristics; positive affect (PA) represents an affective response during AI-assisted learning; perceived usefulness (PU) represents a cognitive evaluation of AI’s learning value; cognitive absorption (CA) reflects deep engagement in AI interaction; mood regulation (MR) captures AI-based regulation of learning-related emotion and pressure; academic stress (AS) represents a contextual pressure; and problematic reliance on generative AI is the outcome. Because inhibitory control, cue reactivity, craving, and longitudinal reinforcement processes were not directly measured, the study applies selected I-PACE components rather than testing the full process model.

Perceived Usefulness and Cognitive Absorption are used to specify two components within this I-PACE-based explanation rather than to introduce separate competing theoretical frameworks. Davis (1989) defined Perceived Usefulness as the degree to which individuals believe that using a technology improves task performance. In this study, PU represents students’ cognitive evaluation of generative AI’s learning value. Agarwal and Karahanna (2000) conceptualized Cognitive Absorption as deep involvement in technology use, characterized by focused attention, perceived control, curiosity, and temporal dissociation. In the present model, CA represents the use-process component of deep engagement during generative AI interaction. Self-Efficacy theory, Need for Cognition theory, and stress–coping theory further support the roles of SE, NC, and AS in explaining students’ AI-related learning behaviors (Bandura, 1986; Cacioppo and Petty, 1982; Lazarus and Folkman, 1984).

### 2.2 Positive affect

Positive Affect (PA) refers to positive emotional experiences such as enjoyment, ease, satisfaction, and a sense of achievement. In generative AI-supported learning, students may experience progress, efficiency, and control when AI provides immediate explanations, writing support, problem-solving cues, or feedback revision. Within the I-PACE model, Positive Affect can be understood as an affective response that reinforces repeated use (Brand et al., 2016, 2019, 2025). In a related study on teachers’ AI addiction, Du et al. (2026) included Positive Affect in an I-PACE-based model, suggesting that positive experiences during AI use may be involved in addiction-related mechanisms.

Positive Affect may first influence Mood Regulation (MR). MR refers to students’ use of generative AI to reduce learning frustration, alleviate task-related anxiety, obtain immediate feedback, and maintain their learning state. When students repeatedly experience ease, achievement, or support during AI-assisted learning, generative AI may become more than a task-completion tool; it may also become a resource for regulating academic stress and restoring a sense of control. This path is grounded in the I-PACE logic of affective reinforcement and the present learning context. Therefore:

*H1a*: Positive Affect is positively associated with Mood Regulation.

Positive Affect may also shape students’ evaluation of AI’s usefulness. If students experience smooth interaction, rapid task feedback, usable suggestions, or visible learning progress when using generative AI, they may perceive the tool as more effective and valuable for learning. This is consistent with the I-PACE assumption that affective responses can influence cognitive evaluations (Brand et al., 2016, 2019, 2025). Therefore:

*H1b*: Positive Affect is positively associated with Perceived Usefulness.

Positive Affect may also be directly associated with problematic reliance on generative AI. If students repeatedly experience enjoyment, efficiency gains, or satisfaction during AI-supported learning, these experiences may reinforce a preference for AI assistance. Over time, students may begin to seek AI support before attempting independent work, increasing excessive reliance on AI outputs and feedback. Because direct student-focused evidence for this specific PA–GA path remains limited, the hypothesis is primarily based on the I-PACE logic of positive reinforcement and the present research context. Therefore:

*H1c*: Positive Affect is positively associated with problematic reliance on generative AI.

### 2.3 Self-efficacy

Self-Efficacy (SE) refers to individuals’ beliefs about their capability to perform specific tasks (Bandura, 1986). In this study, SE refers to students’ belief that they can effectively use generative AI through prompt formulation, output evaluation, iterative revision, and task integration to complete learning tasks. This construct differs from academic Self-Efficacy. Academic Self-Efficacy concerns students’ confidence in their academic ability, whereas SE concerns students’ perceived capability to use AI tools effectively.

Self-Efficacy may influence students’ tendency to use AI for Mood Regulation. Students who feel capable of using generative AI are more likely to treat it as a controllable learning resource when they face difficult tasks, time pressure, or insufficient feedback. They may use AI to seek explanations, decompose tasks, revise ideas, and reduce uncertainty. This path is based on Self-Efficacy theory, the I-PACE model, and the present research context. Therefore:

*H2a*: Self-Efficacy is positively associated with Mood Regulation.

Self-Efficacy may also affect Perceived Usefulness. The practical value of generative AI depends not only on the technology itself but also on whether students can formulate effective prompts, evaluate output quality, and transform AI responses into learning outcomes. Students with higher SE are more likely to obtain useful AI feedback in real learning tasks, which may strengthen their perception of AI’s value. Therefore:

*H2b*: Self-Efficacy is positively associated with Perceived Usefulness.

SE may also facilitate Cognitive Absorption. In generative AI interaction, deep engagement depends partly on whether students can control the direction of dialogue, understand AI feedback, and use it to advance academic tasks. Students with higher SE are more likely to ask clear questions, evaluate AI responses, and continue iterative revision. This process may strengthen perceived control and focused attention, both of which are central to Cognitive Absorption (Agarwal and Karahanna, 2000). Therefore:

*H2c*: Self-Efficacy is positively associated with Cognitive Absorption.

### 2.4 Need for cognition

Need for Cognition (NC) refers to an individual’s tendency to engage in effortful thinking, explore problems, and process information deeply (Cacioppo and Petty, 1982). Students with high NC are often less satisfied with superficial answers and more inclined to compare explanations, ask follow-up questions, and seek deeper cognitive support.

Generative AI can provide explanations, comparisons, expansions, and feedback, making it potentially valuable for students with high NC. Rather than using AI merely to finish tasks quickly, these students may use generative AI to test viewpoints, expand ideas, compare solutions, and examine alternative explanations. Such use may increase their perception of AI’s cognitive support value. However, students with high NC may also be more critical of AI-generated content, making this relationship an empirical question. Therefore:

*H3*: Need for Cognition is positively associated with Perceived Usefulness.

### 2.5 Mood regulation

Mood Regulation (MR) refers to students’ tendency to use generative AI to obtain explanations, reassurance, task decomposition, writing suggestions, or problem-solving cues when they experience learning difficulties, academic pressure, or negative emotions. It is not a general emotional ability; rather, it captures AI-based emotional and academic stress regulation.

The I-PACE model emphasizes compensatory use and reinforcement mechanisms in the development of problematic technology use (Brand et al., 2016, 2019, 2025). Stress–coping theory also suggests that individuals seek coping resources when perceived demands exceed available resources (Lazarus and Folkman, 1984). In academic settings, generative AI can provide immediate explanations, task support, and text generation. If students repeatedly rely on AI to restore their learning state under pressure or frustration, generative AI may shift from being a cognitive tool to becoming a regulatory resource. Therefore:

*H4*: Mood Regulation is positively associated with problematic reliance on generative AI.

### 2.6 Perceived usefulness and cognitive absorption

Perceived Usefulness (PU) has a dual role in generative AI-supported learning. On the one hand, it explains why students incorporate AI into learning activities; on the other hand, it may also explain how ordinary use is associated with problematic reliance on generative AI. Davis (1989) argued that users are more likely to accept and continue using a technology when they believe it improves task performance. In the context of generative AI, students who believe that AI improves efficiency, reduces task burden, provides usable explanations, or supports academic performance may be more likely to rely on it.

Prior studies on AI dependency and generative AI perceptions also support this logic. Chan and Zhou (2023) developed an expectancy-value-based instrument for measuring students’ perceptions of generative AI, highlighting the relevance of perceived value and perceived cost in students’ AI-related evaluations. Zhang et al. (2024) examined performance expectations in relation to AI dependency among university students. Zhong et al. (2024) showed that personal attributes and psychological mechanisms are associated with AI dependency in higher education. In a related study on teachers’ AI addiction, Du et al. (2026) treated Perceived Usefulness as an important cognitive factor in an I-PACE-based explanation of addiction-related AI use. Therefore:

*H5a*: Perceived Usefulness is positively associated with problematic reliance on generative AI.

PU may also promote Cognitive Absorption. When students believe that generative AI is valuable for writing, reviewing, concept understanding, or problem solving, they are more likely to invest attention and time in multi-turn questioning, output revision, and task advancement. This study places PU before CA because it focuses on how students’ evaluation of AI’s learning value encourages more sustained interaction. Therefore:

*H5b*: Perceived Usefulness is positively associated with Cognitive Absorption.

Cognitive Absorption (CA) may itself be associated with problematic reliance on generative AI. Agarwal and Karahanna (2000) described CA as deep involvement in technology use, including focused attention, control, curiosity, and temporal dissociation. Generative AI allows students to continue asking follow-up questions, revising outputs, and expanding ideas around the same academic task. Such sustained engagement may strengthen preference for AI-supported learning environments. In a related study on teachers’ AI addiction, Du et al. (2026) identified cognitive absorption as an important factor in addiction-related generative AI use. Therefore:

*H6*: Cognitive Absorption is positively associated with problematic reliance on generative AI.

### 2.7 Academic stress

Academic Stress (AS) arises from assignments, examinations, papers, grade competition, time management, and future academic or career expectations. According to stress–coping theory, individuals tend to seek coping strategies when task demands exceed their perceived resources (Lazarus and Folkman, 1984). Generative AI can generate answers, explain concepts, provide writing structures, and support exam review in real time, making it attractive as an academic coping resource.

Zhang et al. (2024) incorporated Academic Stress into their study of AI dependency and identified it as an important factor in problematic AI use among university students. The I-PACE model also suggests that negative reinforcement and compensatory use may contribute to addiction-related behavior (Brand et al., 2016, 2019, 2025). When students repeatedly use generative AI to cope with insufficient time, task anxiety, or uncertainty about their own capability, AI may become a stable resource under academic pressure. Therefore:

*H7*: Academic Stress is positively associated with problematic reliance on generative AI.

### 2.8 Research model and configurational research question

Based on the above theoretical reasoning, this study develops a research model of university students’ problematic reliance on generative AI. PLS-SEM is used to estimate the associations among PA, SE, NC, PU, CA, MR, AS, and GA. Specifically, PA is linked to MR, PU, and GA; SE is linked to MR, PU, and CA; NC is linked to PU; MR is linked to GA; PU is linked to GA and CA; CA is linked to GA; and AS is linked to GA.

In addition to these linear associations, problematic reliance on generative AI may also be associated with combinations of psychological and contextual conditions. Therefore, fsQCA is used to examine configurations associated with high GA (Fiss, 2011; Pappas and Woodside, 2021; Ragin, 2008; Schneider and Wagemann, 2012). The study proposes the following research question:

RQ1: Among university students, how do Self-Efficacy, Need for Cognition, Positive Affect, Perceived Usefulness, Cognitive Absorption, Mood Regulation, and Academic Stress combine configurationally in association with high problematic-reliance membership?

Because the empirical analysis also reports non-high GA configurations, these results are treated as an additional asymmetric analysis rather than as the primary research question (Figure 1).

**Figure 1:**
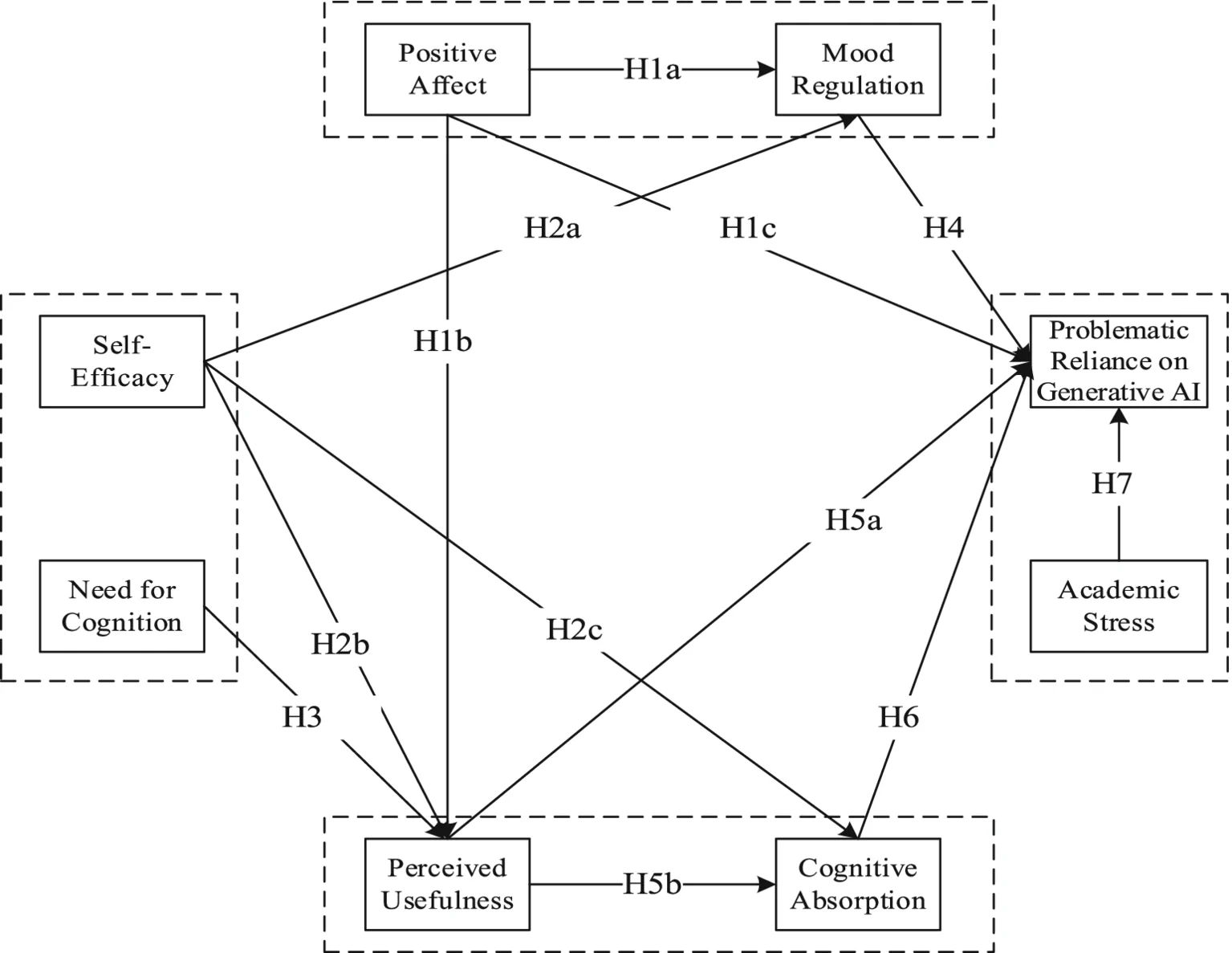
Research framework and hypotheses.

## 3 Materials and methods

### 3.1 Data collection

The participants were adult university students. Data were collected and participants were recruited through Wenjuanxing, an online survey platform, between 26 January 2026 and 30 April 2026, using a non-probability procedure; no probabilistic sampling frame was used. Participation was voluntary, and an electronic informed-consent statement was presented at the beginning of the survey. Respondents were informed that they could withdraw at any time and that the collected data would be used only for academic research. A total of 289 questionnaires were submitted. Four respondents who reported that they had never used generative AI were excluded, leaving 285 responses. Twenty-eight responses were then excluded because the respondents failed the attention-check item, leaving 257 responses. A further 15 responses were excluded because the same response option was selected for all 27 substantive scale items, indicating a straight-line response pattern. The final analytic sample therefore comprised 242 valid responses. Information on participants’ institutions and fields of study was not collected. Consequently, the number of institutions represented, the disciplinary composition of the sample, and the extent to which the sample may have been concentrated within a particular institution or field of study cannot be determined (Table 1).

**Table 1:** Demographic profile and generative AI use characteristics of participants (*N* = 242).

Variable	Category	Frequency	Percentage
Gender	Male	132	54.55%
	Female	110	45.45%
Grade	Freshman	30	12.40%
	Sophomore	74	30.58%
	Junior	55	22.73%
	Senior	34	14.05%
	First-year postgraduate student	19	7.85%
	Second-year postgraduate student	24	9.92%
	Third-year postgraduate student or above	6	2.48%
Duration of generative AI use	Less than 1 month	26	10.74%
	1–3 months	7	2.89%
	3–6 months	9	3.72%
	6–12 months	36	14.88%
	More than 1 year	164	67.77%
Frequency of generative AI use	Almost never	3	1.24%
	1–3 times per month	75	30.99%
	1–3 times per week	89	36.78%
	Almost every day	50	20.66%
	Several times per day	25	10.33%
Frequently used generative AI tools (multiple choice)	ChatGPT	126	52.07%
	Claude	65	26.86%
	Wenxin Yiyan	40	16.53%
	Tongyi Qianwen	33	13.64%
	Kimi	36	14.88%
	DeepSeek	127	52.48%
	Doubao	28	11.57%
	Other	30	12.40%
Main purposes of generative AI use (multiple choice)	Homework/problem solving	145	59.92%
	Thesis/literature review	112	46.28%
	Programming	128	52.89%
	Language learning	38	15.70%
	Presentation/report preparation	38	15.70%
	Exam preparation	12	4.96%
	Other	145	59.92%

### 3.2 Measurement instruments

All constructs were measured using a five-point Likert scale ranging from 1 = strongly disagree to 5 = strongly agree. The questionnaire included eight contextualized constructs: positive affect (PA), generative-AI use self-efficacy (SE), need for cognition (NC), perceived usefulness (PU), cognitive absorption (CA), AI-based mood regulation (MR), academic stress (AS), and problematic reliance on generative AI. GA was retained as the statistical code for problematic reliance on generative AI and was operationalized as a non-clinical, self-reported pattern reflected in perceived overuse, difficulty limiting use, interference with independent task completion, and unease or frustration when generative AI was unavailable.

The contextual item wording for SE, NC, MR, PA, PU, CA, and GA was adapted directly from the teacher-focused generative AI instrument reported by Du et al. (2026) and recontextualized for university students’ learning activities. Du et al. (2026) originally grounded these measures in Wang and Chuang’s (2024) artificial intelligence self-efficacy scale, Lord and Putrevu’s (2006) work on need for cognition, Hutchison and Gunthert’s (2013) work on automatic mood regulation beliefs, Joshanloo’s (2017) positive affect measure, and related technology-use research. In the present study, these labels refer to context-specific AI-related beliefs and experiences rather than unrestricted general traits.

More specifically, PU captures students’ evaluation of generative AI’s learning value and was conceptually grounded in perceived-usefulness research (Davis, 1989; Zou et al., 2023). CA focuses on focused engagement and temporal dissociation during generative AI interaction (Agarwal and Karahanna, 2000; Bozoglan et al., 2014). AS was adapted from Kohn and Frazer’s (1986) academic stress scale. The four outcome items were adapted from the construct labeled “Generative AI Addiction” in Du et al. (2026), informed by research on problematic conversational AI use and AI dependency (Hu et al., 2023; Zhang et al., 2024; Zhong et al., 2024). In the present study, the more conservative label “problematic reliance on generative AI” is used because the measure is not intended to establish a clinical diagnosis. This terminological revision does not alter the item wording, response scale, scoring procedure, structural model, fsQCA calibration, or statistical estimates. This source chain is reported to distinguish the immediate adaptation source from the original conceptual foundations.

The questionnaire was administered in Chinese. The adapted items were translated and reviewed for semantic consistency before data collection. The English wording of the measurement items is provided in Appendix A.

### 3.3 Data analysis method

This study used PLS-SEM and fsQCA to examine psychological and academic factors associated with problematic reliance on generative AI. PLS-SEM was conducted using SmartPLS 4.0 and the current 12-path structural model. The measurement model was evaluated using indicator loadings, Cronbach’s alpha, composite reliability, average variance extracted, the Fornell–Larcker criterion, HTMT ratios, and outer-model VIF values; the structural model was evaluated using path coefficients, specific indirect associations, R^2^, adjusted R^2^, and inner-model VIF values (Chin, 1998; Hair et al., 2019, 2022). Statistical significance was assessed using nonparametric bootstrapping with 5,000 resamples, two-tailed testing, a significance level of 0.05, percentile bootstrap confidence intervals, and a fixed random seed. A sample-size sensitivity analysis was conducted using the inverse square root method (Kock and Hadaya, 2018). With *N* = 242, α = 0.05, and 80% power, the minimum detectable absolute standardized path coefficient was approximately 0.160; smaller associations were therefore interpreted cautiously. Potential common method bias was assessed separately using full-collinearity VIF values (Kock, 2015); values below 3.3 were interpreted as indicating that the results did not show severe common method bias, although this diagnostic was not treated as completely ruling it out. Predictive relevance was assessed using blindfolding with an omission distance of 7, with Q^2^ values greater than zero interpreted as indicating predictive relevance for the corresponding endogenous construct. fsQCA was conducted using fsQCA 4.1 to examine whether high GA and its set-theoretic complement were associated with different configurations of psychological and academic conditions (Fiss, 2011; Pappas and Woodside, 2021; Ragin, 2008; Schneider and Wagemann, 2012). This analytical strategy allowed the study to examine both average model-based associations and configurational patterns.

## 4 Results

### 4.1 Measurement model analysis

PLS-SEM was used to evaluate both the measurement model and the structural model (Hair et al., 2019, 2022). The measurement model was assessed using indicator loadings, reliability, convergent validity, and discriminant validity. Reliability was examined using Cronbach’s alpha and composite reliability (CR), while convergent validity was assessed using the average variance extracted (AVE). As reported in Table 2, indicator loadings ranged from 0.824 to 0.889, and all indicators met the recommended loading criterion. Cronbach’s alpha ranged from 0.803 to 0.881, CR ranged from 0.883 to 0.918, and AVE ranged from 0.712 to 0.771. These values exceeded the commonly recommended thresholds, indicating acceptable reliability and convergent validity (Hair et al., 2019, 2022; Nunnally and Bernstein, 1994). Outer-model VIF values ranged from 1.676 to 2.417, indicating that indicator-level collinearity was not a substantive concern. These values were evaluated separately from the inner-model and full-collinearity VIF values reported below. Discriminant validity was evaluated using the Fornell–Larcker criterion and HTMT. The Fornell–Larcker criterion compares the square root of AVE with inter-construct correlations (Fornell and Larcker, 1981), whereas HTMT provides an additional criterion for assessing discriminant validity in variance-based structural equation modeling (Henseler et al., 2015). As shown in Table 3, the diagonal values ranged from 0.844 to 0.878, and all HTMT values were below 0.85, supporting discriminant validity.

**Table 2:** Measurement model: indicator loadings, reliability, and convergent validity.

Construct	Mean	Factor loading	Outer VIF	α	CR	AVE
Self-efficacy (SE)	3.409	0.880	2.082	0.833	0.900	0.749
	3.388	0.861	1.916			
	3.372	0.855	1.825			
Need for cognition (NC)	3.421	0.889	2.292	0.852	0.910	0.771
	3.393	0.870	2.063			
	3.442	0.876	1.987			
Perceived usefulness (PU)	3.467	0.871	2.417	0.881	0.918	0.736
	3.467	0.867	2.352			
	3.521	0.832	2.024			
	3.380	0.862	2.377			
Cognitive absorption (CA)	3.364	0.859	1.917	0.846	0.907	0.764
	3.298	0.876	2.073			
	3.417	0.887	2.133			
Positive affect (PA)	3.521	0.851	1.875	0.834	0.900	0.750
	3.529	0.866	1.965			
	3.459	0.881	1.962			
Mood regulation (MR)	3.500	0.837	1.805	0.803	0.883	0.716
	3.450	0.842	1.713			
	3.483	0.859	1.676			
Problematic reliance on generative AI (GA)	3.347	0.860	2.053	0.866	0.908	0.712
	3.248	0.838	2.078			
	3.277	0.847	2.202			
	3.264	0.830	1.979			
Academic stress (AS)	3.347	0.857	2.143	0.872	0.912	0.722
	3.368	0.824	2.068			
	3.347	0.854	2.406			
	3.405	0.863	2.310			

CR = composite reliability; AVE = average variance extracted. Outer VIF values refer to indicator-level collinearity diagnostics and are distinct from inner-model and full-collinearity VIF values. The complete questionnaire items are provided in Appendix A. All items were measured using a five-point Likert scale.

**Table 3:** Discriminant validity assessment.

Construct	Square Root of AVE								HTMT						
	SE	NC	PU	CA	PA	MR	GA	AS	SE	NC	PU	CA	PA	MR	GA
SE	**0.866**														
NC	0.362	0.**878**							0.430						
PU	0.453	0.409	**0.858**						0.528	0.469					
CA	0.384	0.320	0.417	**0.874**					0.457	0.375	0.483				
PA	0.465	0.294	0.470	0.328	**0.866**				0.561	0.351	0.545	0.393			
MR	0.407	0.301	0.378	0.338	0.417	**0.846**			0.493	0.359	0.443	0.404	0.499		
GA	0.315	0.301	0.476	0.395	0.321	0.302	**0.844**		0.371	0.349	0.539	0.464	0.374	0.361	
AS	0.390	0.288	0.391	0.433	0.364	0.309	0.435	**0.850**	0.454	0.333	0.441	0.506	0.422	0.363	0.493

The left panel reports the Fornell–Larcker criterion, with the square root of AVE on the diagonal and inter-construct correlations below the diagonal. The right panel reports HTMT ratios. All HTMT values were below 0.85. Bold diagonal values in the left panel are the square roots of the average variance extracted (AVE).

### 4.2 Structural model analysis

The structural model was evaluated using explanatory power, structural collinearity, a full-collinearity diagnostic for potential common method bias, and blindfolding-based predictive relevance. The R^2^ values were 0.222 for CA, 0.322 for GA, 0.232 for MR, and 0.339 for PU; the corresponding adjusted R^2^ values were 0.216, 0.308, 0.225, and 0.331. Here, GA is retained as the statistical code for problematic reliance on generative AI. These values indicate modest explanatory power across the endogenous constructs. Inner-model VIF values ranged from 1.178 to 1.504, indicating that collinearity among the structural predictors was not a substantive concern. These values were distinct from the outer-model VIF values, which ranged from 1.676 to 2.417. Full-collinearity VIF values were 1.481 for AS, 1.459 for CA, 1.481 for GA, 1.383 for MR, 1.307 for NC, 1.533 for PA, 1.737 for PU, and 1.570 for SE. All values were below 3.3. The results therefore did not indicate severe common method bias. Nevertheless, because all variables were measured using the same self-report questionnaire at one time point, common method bias cannot be completely ruled out. Blindfolding was conducted using an omission distance of 7. The Q^2^ values were 0.156 for MR, 0.240 for PU, 0.165 for CA, and 0.221 for GA. All four Q^2^ values were greater than zero, indicating predictive relevance for each endogenous construct (Figure 2).

**Figure 2:**
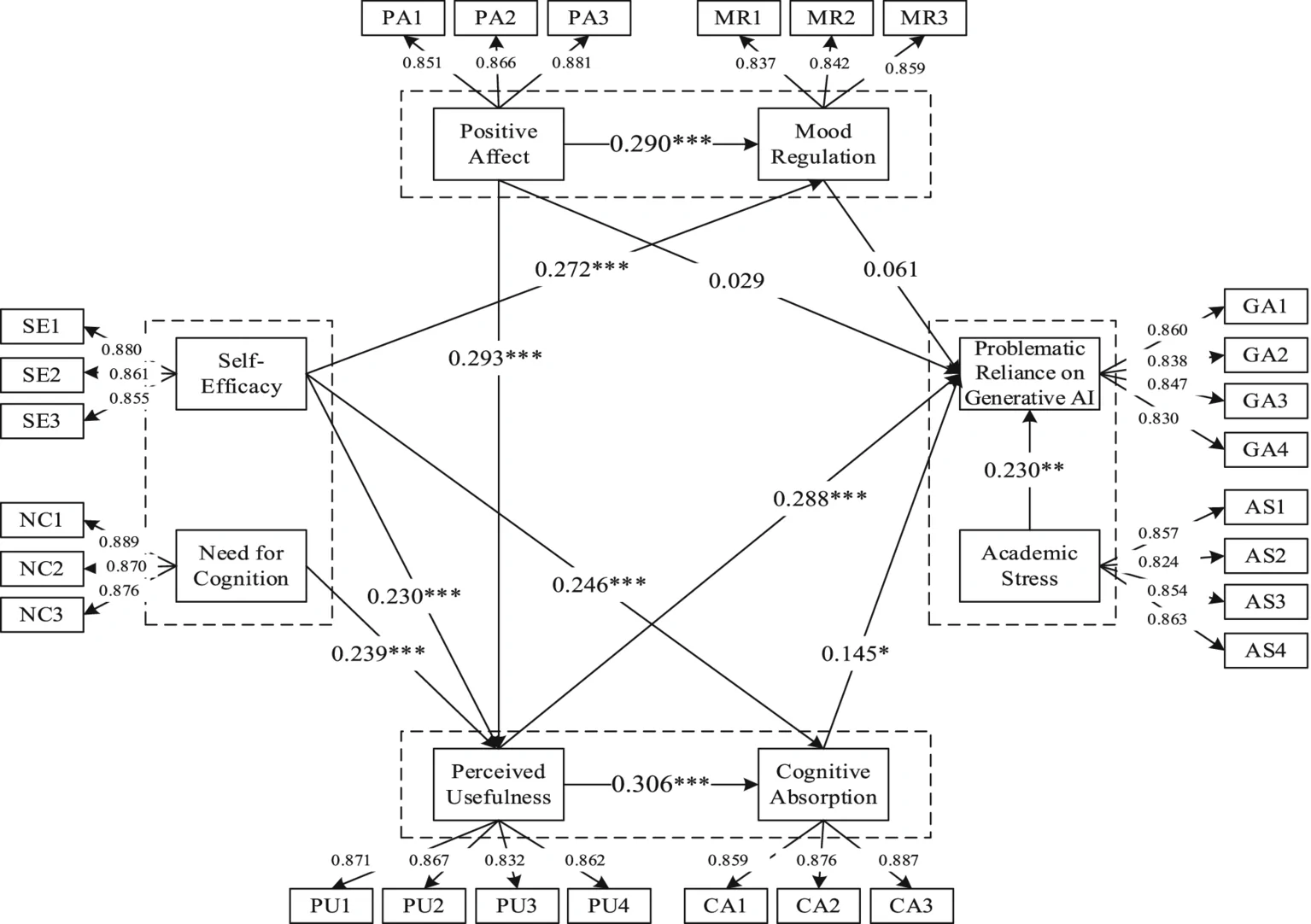
Structural model results. Standardized path coefficients and indicator loadings are shown. The PA → GA and MR → GA paths are not statistically significant. ***p < 0.001, **p < 0.01, *p < 0.05.

### 4.3 Direct associations

The direct associations are presented in Table 4. Positive affect (PA) was positively associated with AI-based mood regulation (MR; β = 0.290, t = 4.415, *p* < 0.001), supporting H1a, and with perceived usefulness (PU; β = 0.293, t = 4.415, *p* < 0.001), supporting H1b. In contrast, the association between PA and GA was not statistically significant (β = 0.029, t = 0.406, *p* = 0.685); therefore, H1c was not supported.

**Table 4:** Direct associations.

Hypothesis	Path	Beta	Standard deviation (std)	t value	*p* value	Results
H1a	PA → MR	0.290	0.066	4.415	<0.001	Supported
H1b	PA → PU	0.293	0.066	4.415	<0.001	Supported
H1c	PA → GA	0.029	0.071	0.406	0.685	Not supported
H2a	SE → MR	0.272	0.064	4.280	<0.001	Supported
H2b	SE → PU	0.230	0.067	3.414	<0.001	Supported
H2c	SE → CA	0.246	0.065	3.777	<0.001	Supported
H3	NC → PU	0.239	0.060	3.996	<0.001	Supported
H4	MR → GA	0.061	0.065	0.938	0.348	Not supported
H5a	PU → GA	0.288	0.078	3.683	<0.001	Supported
H5b	PU → CA	0.306	0.064	4.809	<0.001	Supported
H6	CA → GA	0.145	0.071	2.048	0.041	Supported
H7	AS → GA	0.230	0.071	3.223	0.001	Supported

β values are standardized path coefficients. PA = Positive Affect; SE = Self-Efficacy; NC = Need for Cognition; PU = Perceived Usefulness; CA = Cognitive Absorption; MR = Mood Regulation; AS = Academic Stress; GA = problematic reliance on generative AI (statistical code). ***p < 0.001, **p < 0.01, *p < 0.05.

Generative-AI use self-efficacy (SE) was positively associated with MR (β = 0.272, t = 4.280, *p* < 0.001), supporting H2a; with PU (β = 0.230, t = 3.414, *p* < 0.001), supporting H2b; and with cognitive absorption (CA; β = 0.246, t = 3.777, *p* < 0.001), supporting H2c.

Need for cognition (NC) was positively associated with PU (β = 0.239, t = 3.996, *p* < 0.001), supporting H3. By contrast, MR was not significantly associated with GA (β = 0.061, t = 0.938, *p* = 0.348); thus, H4 was not supported.

Among the direct predictors of GA, PU was positively associated with GA (β = 0.288, t = 3.683, *p* < 0.001), supporting H5a, and with CA (β = 0.306, t = 4.809, *p* < 0.001), supporting H5b. CA showed a statistically significant but relatively weak positive association with GA (β = 0.145, t = 2.048, *p* = 0.041), supporting H6. Academic stress (AS) was also positively associated with GA (β = 0.230, t = 3.223, *p* = 0.001), supporting H7. Given the cross-sectional design, these coefficients indicate model-based associations rather than established causal effects.

Overall, the direct-association analysis shows a clear contrast between supported and unsupported pathways. PU, CA, and AS were directly associated with GA, whereas PA and MR did not show significant direct associations with GA.

### 4.4 Specific indirect associations

Specific indirect associations were examined using bootstrapping and are reported in Table 5. Several paths involving PU reached statistical significance: PA → PU → GA (β = 0.084, t = 2.790, *p* = 0.005), SE → PU → CA (β = 0.070, t = 3.032, *p* = 0.002), SE → PU → GA (β = 0.066, t = 2.531, *p* = 0.011), NC → PU → CA (β = 0.073, t = 2.912, *p* = 0.004), NC → PU → GA (β = 0.069, t = 2.545, *p* = 0.011), and PA → PU → CA (β = 0.090, t = 3.054, *p* = 0.002). Because the data are cross-sectional, these estimates indicate statistical indirect associations rather than established temporal mediation.

**Table 5:** Specific indirect associations.

Indirect path	β	t	*p*	Decision
PA → PU → GA	0.084	2.790	0.005	Supported
SE → PU → CA	0.070	3.032	0.002	Supported
SE → PU → GA	0.066	2.531	0.011	Supported
PA → MR → GA	0.018	0.882	0.378	Not supported
SE → MR → GA	0.017	0.890	0.373	Not supported
PU → CA → GA	0.044	1.866	0.062	Not supported
SE → CA → GA	0.036	1.770	0.077	Not supported
NC → PU → CA → GA	0.011	1.578	0.115	Not supported
SE → PU → CA → GA	0.010	1.742	0.082	Not supported
PA → PU → CA → GA	0.013	1.602	0.109	Not supported
NC → PU → CA	0.073	2.912	0.004	Supported
NC → PU → GA	0.069	2.545	0.011	Supported
PA → PU → CA	0.090	3.054	0.002	Supported

β values are standardized indirect associations. PA = Positive Affect; SE = Self-Efficacy; NC = Need for Cognition; PU = Perceived Usefulness; CA = Cognitive Absorption; MR = Mood Regulation; AS = Academic Stress; GA = problematic reliance on generative AI (statistical code). Decisions are based on two-tailed *p* values.

In contrast, the MR-related indirect associations were not significant. The indirect association of PA with GA through MR was not supported (β = 0.018, t = 0.882, *p* = 0.378), and the indirect association of SE with GA through MR was also not supported (β = 0.017, t = 0.890, *p* = 0.373). These findings are consistent with the non-significant direct association of MR with GA.

The CA-mediated indirect associations involving GA did not reach conventional significance levels. Specifically, PU → CA → GA was not supported (β = 0.044, t = 1.866, *p* = 0.062), and SE → CA → GA was not supported (β = 0.036, t = 1.770, *p* = 0.077). The chained indirect associations NC → PU → CA → GA (β = 0.011, t = 1.578, *p* = 0.115), SE → PU → CA → GA (β = 0.010, t = 1.742, *p* = 0.082), and PA → PU → CA → GA (β = 0.013, t = 1.602, *p* = 0.109) were also not supported.

Taken together, PU appeared in the main statistically supported indirect associations, whereas MR-related and CA-related indirect paths to GA were not statistically supported. These patterns should be interpreted as model-based associations rather than confirmed mediation mechanisms.

### 4.5 fsQCA analysis

#### 4.5.1 Variable selection and calibration

To further examine the configurational patterns associated with problematic reliance on generative AI, this study conducted fuzzy-set qualitative comparative analysis (fsQCA). fsQCA is suitable for examining conjunctural patterns, equifinality, and set-theoretic asymmetry (Fiss, 2011; Pappas and Woodside, 2021; Ragin, 2008; Schneider and Wagemann, 2012). The outcome variable was problematic reliance on generative AI (GA), and the condition variables were Self-Efficacy (SE), Need for Cognition (NC), Perceived Usefulness (PU), Cognitive Absorption (CA), Positive Affect (PA), Mood Regulation (MR), and Academic Stress (AS).

The raw scores were calibrated into fuzzy-set membership scores using the direct calibration method. The 95th percentile, 50th percentile, and 5th percentile were used as the anchors for full membership, crossover point, and full non-membership, respectively. To avoid ambiguity at the maximum point of fuzziness, calibrated values equal to 0.5 were replaced with 0.501. The calibration anchors are reported in Table 6.

**Table 6:** Calibration anchors for fsQCA.

Type	Variable	Full membership	Crossover point	Full non-membership
Outcome variable	GA	4.99	3.25	1.50
Condition variable	SE	5.00	3.33	2.00
	NC	5.00	3.67	1.67
	PU	5.00	3.50	1.75
	CA	5.00	3.33	1.67
	PA	5.00	3.67	1.67
	MR	5.00	3.33	1.68
	AS	5.00	3.50	1.75

### 4.6 Necessity analysis

A necessity analysis was first conducted for both high problematic-reliance membership (GA) and its set-theoretic complement (~GA). In fsQCA, a condition is generally considered necessary when its consistency reaches or exceeds 0.900 (Ragin, 2008; Schneider and Wagemann, 2012). The results showed that neither the presence nor the absence of any single condition reached this threshold for GA. The same conclusion was obtained for ~GA. Therefore, neither high problematic-reliance membership nor its complement can be explained by a single necessary condition. The detailed necessity analysis results are presented in Table 7.

**Table 7:** Necessity analysis results.

Condition variable	GA		~GA	
	Consistency	Coverage	Consistency	Coverage
SE	0.716686	0.736998	0.571261	0.557494
~SE	0.569689	0.583361	0.730503	0.709887
NC	0.691268	0.748161	0.569428	0.584865
~NC	0.616433	0.601370	0.754808	0.698812
PU	0.769841	0.775140	0.549824	0.525377
~PU	0.528623	0.553045	0.764678	0.759208
CA	0.746686	0.756335	0.564506	0.542641
~CA	0.548476	0.570283	0.746516	0.736614
PA	0.729516	0.748963	0.579120	0.564238
~PA	0.575552	0.590329	0.742341	0.722571
MR	0.768681	0.720762	0.639238	0.568822
~MR	0.540156	0.612060	0.686195	0.737888
AS	0.729491	0.778409	0.551903	0.558880
~AS	0.586602	0.579734	0.781175	0.732659

A condition is generally considered necessary when consistency is equal to or greater than 0.900. GA denotes high membership in problematic reliance on generative AI; ~GA denotes its set-theoretic complement rather than a separately classified low-reliance or clinical group.

### 4.7 Sufficiency analysis for high problematic-reliance membership (GA)

After the necessity analysis, a truth table was constructed to identify sufficient configurations for high GA. The case frequency threshold was set to 2, the consistency threshold was set to 0.80, and the PRI threshold was set to 0.75. These criteria are commonly used to evaluate configurational consistency and empirical relevance in fsQCA applications (Fiss, 2011; Pappas and Woodside, 2021; Ragin, 2008). The intermediate solution identified four configurations associated with high GA, with overall solution coverage of 0.506 and overall solution consistency of 0.942. These results describe multiple, partly overlapping conjunctural patterns in the observed sample. The detailed high-GA configurations are reported in Table 8.

**Table 8:** Configurations for high problematic-reliance membership (GA).

Condition variable	HGA1		HGA2	
	HGA1a	HGA1b	HGA2a	HGA2b
SE	•		●	●
NC		•	●	●
PU	●	●		•
CA	●	●	●	●
PA		•	•	•
MR	●	●	•	
AS	●	●	●	●
Raw coverage	0.457226	0.404692	0.387538	0.393578
Unique coverage	0.0783145	0.02578	0.00862569	0.0146658
Consistency	0.957337	0.958767	0.950217	0.959175
Solution coverage	0.506298			
Solution consistency	0.941699			

● = core condition present; • = peripheral condition present; ⊗ = core condition absent;⊗ = peripheral condition absent; a blank cell indicates that the condition is not specified in the configuration. Core and peripheral conditions were determined by comparing the parsimonious and intermediate solutions. HGA identifies high-GA configurations; NHGA identifies configurations for ~GA.

### 4.8 Sufficiency analysis for the set-theoretic complement of high problematic-reliance membership (~GA)

This study also examined configurations associated with the set-theoretic complement of high problematic-reliance membership (~GA). This outcome is the fuzzy-set complement of high problematic-reliance membership rather than a separately classified low-reliance or clinical group. The intermediate solution identified seven configurations, with an overall solution coverage of 0.586 and an overall solution consistency of 0.901. The detailed ~GA configurations are reported in Table 9.

**Table 9:** Configurations for the set-theoretic complement of high problematic-reliance membership (~GA).

Condition variable	NHGA1		NHGA2		NHGA3		NHGA4
	NHGA1a	NHGA1b	NHGA2a	NHGA2b	NHGA3a	NHGA3b	
SE		●	⊗	⊗	⊗	●	⊗
NC			⊗	⊗	⊗	⊗	⊗
PU	⊗	⊗	⊗	⊗	⊗	⊗	⊗
CA	⊗	⊗		⊗	⊗	⊗	●
PA	⊗	⊗	⊗	⊗		●	
MR	⊗	⊗			⊗	●	●
AS	⊗		⊗		●	●	⊗
Raw coverage	0.435562	0.308289	0.461462	0.462718	0.297545	0.219716	0.28642
Unique coverage	0.0118812	0.0128996	0.0145884	0.00686562	0.00493073	0.0117964	0.0102602
Consistency	0.942382	0.968462	0.917057	0.927721	0.968831	0.965	0.956036
Solution coverage	0.586452						
Solution consistency	0.901353						

Filled circles (●) indicate the presence of a condition; crossed circles (⊗) indicate its absence; blank cells indicate that the condition is not specified in the configuration. ~GA denotes the set-theoretic complement of high problematic-reliance membership.

### 4.9 Summary of fsQCA results

The fsQCA results provide two main insights. First, no single condition was necessary for either high problematic-reliance membership (GA) or its set-theoretic complement (~GA). Second, both outcome sets were associated with multiple, partly overlapping configurations: four for GA and seven for ~GA. These patterns are consistent with conjunctural relations and equifinality (Fiss, 2011; Ragin, 2008), but they should not be interpreted as longitudinal causal pathways. The fsQCA results complement the PLS-SEM findings by showing how conditions co-occur in the observed sample.

## 5 Discussion

### 5.1 Discussion of PLS-SEM findings

The PLS-SEM results provide a net-association description of university students’ scores for problematic reliance on generative AI (GA). PU, CA, and AS were positively associated with GA, whereas PA and MR were not significantly associated with GA as direct predictors in the specified model. Within this sample, GA was more closely associated with students’ evaluation of AI’s academic value, their depth of engagement with AI interaction, and academic stress than with positive affect or AI-based mood regulation considered in isolation.

The positive association between PU and GA identifies students’ evaluation of generative AI as useful for learning as a relevant correlate of problematic reliance. This finding is consistent with technology-acceptance research and student-focused studies linking perceived value and performance expectations with AI use and dependency (Chan and Zhou, 2023; Davis, 1989; Zhang et al., 2024; Zhong et al., 2024). One possible interpretation is that perceived efficiency and workload reduction encourage reliance; the reverse interpretation, that reliance increases perceived usefulness, cannot be excluded.

CA also showed a significant positive association with GA, but the effect was relatively weak. This finding suggests that deep engagement in AI interaction may be linked to GA, but it should not be overstated as the dominant driver in the present model. Cognitive absorption captures focused attention, perceived control, curiosity, and temporal dissociation in technology use (Agarwal and Karahanna, 2000). In a related study on teachers’ AI addiction, Du et al. (2026) also identified cognitive absorption as an important factor in GA-related excessive reliance. Nevertheless, the relatively weak effect in this study indicates that immersion alone is insufficient to explain GA.

AS was also positively associated with problematic reliance on generative AI, indicating that academic context is relevant alongside technology-related beliefs. Stress–coping theory suggests that students facing high demands may seek resources that reduce task burden, uncertainty, or pressure (Lazarus and Folkman, 1984), and Zhang et al. (2024) likewise linked academic stress with problematic AI use. The present cross-sectional association is consistent with, but does not prove, the interpretation that students under greater academic pressure may rely more heavily on generative AI. The reverse relationship is also plausible. Students reporting greater problematic reliance on generative AI may subsequently experience greater academic stress if reliance is accompanied by weaker independent task-completion skills, postponement of academic work, or last-minute catch-up when generative AI is unavailable or performs poorly. Because academic stress and problematic reliance were measured concurrently, the present data cannot determine whether academic stress precedes problematic reliance, follows from it, or operates reciprocally.

PA and MR were not significantly associated with GA as direct predictors in the specified PLS-SEM model. This result does not demonstrate that positive emotion or AI-based regulation prevents or causes GA; it only indicates that their isolated direct coefficients were not distinguishable from zero in this sample. MR appeared in some fsQCA configurations, showing conditional co-occurrence rather than a hidden causal effect. This cautious interpretation is compatible with the I-PACE view that affective and regulatory processes may operate within broader interactions (Brand et al., 2016, 2019, 2025).

### 5.2 Discussion of fsQCA findings

The fsQCA results extend the PLS-SEM findings by revealing the configurational nature of problematic reliance on generative AI (GA). The necessity analysis showed that no single condition reached the conventional necessity threshold of 0.900. This means that high GA cannot be attributed to any one factor alone. Even conditions that appear important in the structural model, such as PU, CA, and AS, should not be interpreted as necessary conditions. Instead, they should be understood as components that may become important when combined with other psychological and contextual conditions.

The sufficiency analysis identified four configurations associated with high GA, with an overall solution coverage of 0.506 and an overall solution consistency of 0.942. The configurations overlapped substantially, as indicated by several small unique-coverage values; they should therefore not be treated as equally independent empirical routes. The pattern is consistent with the fsQCA concepts of conjunctural causality and equifinality (Fiss, 2011; Pappas and Woodside, 2021; Ragin, 2008; Schneider and Wagemann, 2012).

CA and AS appeared in all four high-GA configurations, while PU appeared in three. Their repeated appearance indicates configurational relevance within these solutions, not individual necessity or a stand-alone causal effect. Because some configurations contributed little unique coverage, interpretation should emphasize the recurring combined pattern rather than assign equal importance to every configuration.

The analysis of ~GA identified seven configurations, with an overall solution coverage of 0.586 and an overall solution consistency of 0.901. The ~GA solutions were not simple reversals of the high-GA solutions, which is consistent with causal asymmetry in set-theoretic analysis (Ragin, 2008; Schneider and Wagemann, 2012). Here, however, ~GA denotes the negation of fuzzy membership in high GA and should not be interpreted as a clinically defined low-addiction group.

### 5.3 Integration of PLS-SEM and fsQCA findings

PLS-SEM and fsQCA provide two complementary descriptions of the same cross-sectional data. PLS-SEM estimates average model-based associations, showing positive associations of PU, CA, and AS with GA and no significant direct associations of PA or MR with GA. fsQCA examines which observed combinations are sufficient for membership in high GA or ~GA. The two approaches address different questions and do not, by themselves, establish temporal or causal mechanisms.

The cross-method comparison shows both convergence and divergence. CA and AS were positively associated with GA in PLS-SEM and recurred in the high-GA configurations, whereas MR was not significantly associated with GA as an isolated net effect but appeared within some configurations. This does not mean that MR has a hidden causal effect; rather, its relevance in fsQCA is conditional on co-occurrence with other conditions. CA likewise showed a relatively weak net association but recurrent configurational presence.

### 5.4 Theoretical implications

This study offers three bounded theoretical contributions. First, it conceptually replicates and extends Du et al.’s (2026) teacher-focused application in a university-student learning context and incorporates academic stress as an additional contextual condition. The findings show how selected personal, affective, cognitive, engagement, regulatory, and academic-pressure variables are associated with GA within an I-PACE-informed framework; they do not constitute a test of the full developmental I-PACE process (Brand et al., 2016, 2019, 2025).

Second, the study treats problematic reliance as conceptually distinct from general AI use, technology acceptance, and ordinary continuance use. Prior work has primarily examined students’ perceptions, acceptance, perceived value, educational use, and academic-integrity concerns (Baek et al., 2024; Chan and Hu, 2023; Chan and Zhou, 2023; Chen et al., 2025; Cotton et al., 2024; Farrokhnia et al., 2024; Kasneci et al., 2023). The present operationalization focuses instead on self-reported overuse, difficulty limiting use, interference with independent task completion, and negative reactions to unavailability, while remaining non-clinical.

Third, the study illustrates how linear and configurational analyses can provide complementary cross-sectional evidence. PLS-SEM estimates average associations among PA, SE, NC, PU, CA, MR, AS, and GA, whereas fsQCA identifies observed combinations associated with high GA and ~GA. These analyses strengthen description of the sample but do not establish longitudinal causal pathways.

### 5.5 Practical implications

The findings suggest several potential implications for higher education, although the cross-sectional data do not test the effectiveness of specific interventions. Universities may wish to distinguish appropriate AI-supported learning from problematic reliance rather than respond through blanket prohibition. Clear AI-use guidelines can help students understand when assistance is appropriate, how it should be disclosed, and which parts of academic work must remain student-generated. Prior research likewise emphasizes addressing academic integrity without relying solely on prohibition (Cotton et al., 2024; Farrokhnia et al., 2024; Kasneci et al., 2023).

Second, institutions may consider integrating AI literacy into academic support. Relevant competencies include evaluating AI outputs, identifying unsupported claims, revising generated content, and using AI for feedback without substituting it for students’ own reasoning. These suggestions are consistent with AI literacy frameworks but were not directly evaluated in the present study (Chen et al., 2025; Long and Magerko, 2020; Ng et al., 2021).

Third, the positive association between academic stress and GA suggests that students experiencing high academic pressure may be a relevant group for further support. Learning support, stress-management resources, writing assistance, and staged task guidance are plausible responses, but their effects on problematic AI reliance require direct evaluation.

Fourth, instructors may consider process-oriented assessment, staged submissions, oral explanations, AI-use reflection logs, and selected in-class activities as ways to make students’ own reasoning more visible. These practices are consistent with the broader academic-integrity literature, but the present data do not demonstrate that they reduce problematic reliance (Cotton et al., 2024; Farrokhnia et al., 2024; Kasneci et al., 2023; Rudolph et al., 2023).

### 5.6 Limitations and future research

Several limitations should be noted. First, the cross-sectional design does not establish temporal order or causality. Although the theoretical model specifies directional paths, reverse or reciprocal relationships are plausible; for example, students who already report greater problematic reliance on generative AI may subsequently perceive it as more useful, become more cognitively absorbed, or experience greater academic stress if reliance is accompanied by weaker independent task-completion skills, postponement of academic work, or last-minute catch-up when generative AI is unavailable or performs poorly. Longitudinal, experimental, or learning-trace designs are needed to examine temporal ordering and reciprocal relationships.

Second, all constructs were measured through the same self-report questionnaire at one time point. The full-collinearity VIF assessment did not indicate severe common method bias, but this diagnostic does not completely rule out common method influences. Recall error, social desirability, and shared measurement context may therefore have affected the observed associations. The measures capture perceived tendencies rather than verified AI-use behavior, clinical diagnosis, or objectively assessed impairment.

Third, participants were recruited through Wenjuanxing using a non-probability online procedure. Information on participants’ institutions and fields of study was not collected. It is therefore not possible to determine the number of institutions represented, the disciplinary composition of the sample, or whether the sample was concentrated within a particular institution or academic field. These limitations restrict population-level generalization and prevent assessment of institutional or disciplinary clustering.

Finally, several measures were substantially contextualized for generative AI-supported learning, so their labels should not be assumed to represent unrestricted general traits without further validation. The fsQCA solutions are also dependent on the selected conditions, calibration anchors, and thresholds, and several configurations show limited unique coverage. Future research should validate the contextualized measures, examine alternative calibrations, incorporate variables such as AI literacy and academic-integrity awareness, and combine surveys with interviews, case studies, or learning-log data.

## 6 Conclusion

This study examined problematic learning-related reliance on generative AI among university students, with GA retained solely as the statistical code and explicitly not as a clinical diagnosis. Using an I-PACE-informed framework, it combined PLS-SEM and fsQCA to examine average associations and configurational patterns involving selected psychological and academic conditions.

The PLS-SEM results indicated that perceived usefulness (PU), cognitive absorption (CA), and academic stress (AS) were positively associated with GA, whereas positive affect (PA) and AI-based mood regulation (MR) did not show significant direct associations. In fsQCA, no single condition reached the necessity threshold; four configurations were associated with high GA and seven with its set-theoretic complement (~GA). These cross-sectional findings indicate recurring combinations of cognitive evaluation, engagement, affective-regulatory conditions, and academic pressure, but they do not establish causal pathways.

The study contributes a university-student conceptual replication and extension of Du et al.’s (2026) teacher-focused model by adding academic stress and configurational analysis. It also distinguishes the measured pattern of problematic reliance from ordinary AI use and technology acceptance. The findings suggest possible directions for AI literacy and academic support, although the effectiveness of specific educational interventions was not tested.

Interpretation should account for the cross-sectional, same-source self-report design; non-probability sampling; possible reverse relationships and common-method variance; substantial contextualization of several measures; and the dependence of fsQCA solutions on calibration and threshold choices. Future longitudinal, experimental, interview-based, and learning-log studies should validate the measures and examine whether the observed associations generalize across institutional, disciplinary, and policy contexts.

## Data Availability

The raw data supporting the conclusions of this article will be made available by the authors, without undue reservation, upon reasonable request.
