# Supplementary material for: Problematic reliance on generative AI among university students: an I-PACE-informed PLS-SEM and fsQCA study
Source: Front Psychol. 2026 Oct 5;17:1946110. doi: 10.3389/fpsyg.2026.1946110 (PMC13677851; doi:10.3389/fpsyg.2026.1946110)
Supplement: Supplementary file 1 [file Supplementary_file_1.docx]

**Appendix A**

**Table A1. Measurement Items.**

| **Construct** | **Item code** | **Item wording** |
| --- | --- | --- |
| Self-Efficacy (SE) | SE1 | I am confident in my ability to use generative AI tools to support learning tasks. |
|  | SE2 | I believe that through generative AI, I can efficiently complete various learning tasks, such as writing, problem solving, and reviewing. |
|  | SE3 | When I encounter learning difficulties, I am confident that I can find solutions through generative AI. |
| Need for cognition (NC) | NC1 | I enjoy exploring how generative AI can help me improve my learning methods and strategies. |
|  | NC2 | I often use generative AI to find more innovative ways to understand or explain complex knowledge. |
|  | NC3 | When I discover new ways of using generative AI in learning, I feel inspired and stimulated. |
| Mood regulation (MR) | MR1 | I use generative AI to reduce pressure from completing assignments, exam preparation, papers, and other learning tasks. |
|  | MR2 | When I feel overwhelmed by learning tasks, I turn to generative AI to reduce the burden. |
|  | MR3 | When I have many tasks to complete, using generative AI helps me proceed with learning tasks more calmly and with greater focus. |
| Positive affect (PA) | PA1 | When I use generative AI to produce valuable learning outcomes, such as high-quality assignments, reports, or ideas for answers, I feel excited. |
|  | PA2 | Using generative AI makes me more motivated to try new learning methods, such as different problem-solving paths, writing frameworks, or review plans. |
|  | PA3 | When I integrate AI-generated content or suggestions into my learning output, I feel a sense of achievement. |

**Appendix A**

**Table A1. Measurement Items (continued).**

| **Construct** | **Item code** | **Item wording** |
| --- | --- | --- |
| Perceived usefulness (PU) | PU1 | Generative AI helps me complete high-quality learning tasks more efficiently, such as assignments, reports, and review materials. |
|  | PU2 | Using generative AI allows me to devote more energy to important learning activities, such as understanding, thinking, and practice, rather than being held back by repetitive work. |
|  | PU3 | Generative AI provides many valuable ideas or resources that I might not have thought of on my own. |
|  | PU4 | I believe generative AI is a useful tool for improving my learning efficiency and output. |
| Cognitive absorption (CA) | CA1 | When I use generative AI to complete learning tasks, I often lose track of time. |
|  | CA2 | When I use generative AI to handle learning tasks, I become so involved that I hardly notice what is happening around me. |
|  | CA3 | When I use generative AI to complete learning content or learning outputs, I feel very focused and engaged. |
| Problematic Reliance on Generative AI (GA) | GA1 | I often feel that I use generative AI more frequently or for longer than I should in learning tasks. |
|  | GA2 | I have tried to limit my use of generative AI, but I find it difficult to do so. |
|  | GA3 | My reliance on generative AI sometimes affects my ability to complete learning tasks independently without it. |
|  | GA4 | When I cannot use generative AI, I feel uneasy or frustrated. |
| Academic stress (AS) | AS1 | I often feel that academic tasks are heavy and that there is never enough time. |
|  | AS2 | I often feel anxious because of deadlines for academic tasks. |
|  | AS3 | I feel unable to cope with the pressure of academic tasks. |
|  | AS4 | Academic pressure often makes me feel tense and uneasy. |
